# Genetic diversity, evolution and drug resistance of *Mycobacterium tuberculosis* lineage 2

**DOI:** 10.3389/fmicb.2024.1384791

**Published:** 2024-05-17

**Authors:** Sabina Atavliyeva, Dana Auganova, Pavel Tarlykov

**Affiliations:** Genomics and Proteomics Core Facility, National Center for Biotechnology, Astana, Kazakhstan

**Keywords:** *Mycobacterium tuberculosis*, phylogeny, tuberculosis, multidrug-resistant, virulence, disease outbreaks

## Abstract

*Mycobacterium tuberculosis* causes a chronic infectious disease called tuberculosis. Phylogenetic lineage 2 (L2) of *M. tuberculosis*, also known as the East Asian lineage, is associated with high virulence, increased transmissibility, and the spread of multidrug-resistant strains. This review article examines the genomic characteristics of the *M. tuberculosis* genome and *M. tuberculosis* lineage 2, such as the unique insertion sequence and spoligotype patterns, as well as MIRU-VNTR typing, and SNP-based barcoding. The review describes the geographical distribution of lineage 2 and its history of origin. In addition, the article discusses recent studies on drug resistance and compensatory mechanisms of *M. tuberculosis* lineage 2 and its impact on the pathogen’s transmissibility and virulence. This review article discusses the importance of establishing a unified classification for lineage 2 to ensure consistency in terminology and criteria across different studies and settings.

## 1 Introduction

*Mycobacterium tuberculosis* (MTB) poses a serious threat to human health, with 10 million people becoming infected with tuberculosis every year. Until the coronavirus (COVID-19) pandemic, TB was the leading cause of death from a single infectious agent, ranking above HIV/AIDS. In 2022, according to the World Health Organization (WHO), tuberculosis remains the second leading cause of death from infectious diseases after COVID-19 and caused almost twice as many deaths as HIV/AIDS (World Health Organization, 2023). Patients with comorbidities such as TB and HIV/AIDS are at significantly higher risk of developing severe forms of COVID-19 (Udoakang et al., 2023). Co-infection with COVID-19 and tuberculosis results in higher mortality than in patients with COVID-19 alone (du Bruyn et al., 2023). HIV also increases the risk of latent tuberculosis progressing to its active form by 26 times (Yang et al., 2022). Thus, co-infection provides synergistic effects to both pathogens, resulting in atypical clinical manifestations of tuberculosis.

Among MTB genetic lineages, lineage 2 (L2), also known as East Asian lineage, stands out as one of the most widely found lineages. This lineage, mainly represented by the Beijing family, plays a significant role in the global epidemiology of tuberculosis, accounting for nearly a quarter of all reported TB cases (Liu et al., 2020). Experimental and clinical data indicate high virulence and increased mutation rates in Beijing strains, highlighting their clinical and epidemiological significance (Guerra-Assunção et al., 2015; Rajwani et al., 2018). Understanding the genetic and phenotypic features of L2, characterized by highly transmissible strains and multidrug resistance, is critical for developing effective strategies for the treatment and control of tuberculosis (Holt et al., 2018; Liu et al., 2018a).

The *Mycobacterium tuberculosis* Beijing genotype was first identified by Van Soolingen et al. (1995) in the mid-1990s in China. The strains differed in the pattern of the IS*6110* insertion sequence with 15–20 copies in the genome and were also distinguished by a peculiar spoligotype, where only spacers from 35 to 43 in the DR locus predominated (Van Soolingen et al., 1995). Strains of this lineage may have originated in East Asia in its northern or southern parts and then spread through China to the rest of the world. It subsequently attracted attention for its ability to cause large epidemics in various parts of the world, especially in Central Asia, Eastern Europe, and Eastern Africa (Pardini et al., 2009; Rutaihwa et al., 2019; Daniyarov et al., 2021). In a short time, both multidrug resistance (MDR) and extensive drug resistance (XDR) were reported to be developed. It is known that mycobacteria have mechanisms of resistance to all anti-tuberculosis drugs that exist today (World Health Organization, 2021).

This article covers current research on the genetic characteristics of MTB lineage 2, highlighting its implications for understanding the epidemiology and diagnosis of tuberculosis. Next, the genomic diversity of lineage 2 is described concerning the phylogeny and classification of sublineages and clusters. In addition, the distribution of these sublineages across geographic regions, with a description of the place and time of origin of lineage 2 is covered. Finally, the latest knowledge on drug resistance of *M. tuberculosis* and the impact of compensatory mutations on the development of high transmissibility and virulence is discussed. This review article aims to provide a state-of-the-art overview of the genomic characterization of lineage 2 strains, identifying key trends, challenges, and prospects for future research.

## 2 Genomic characteristics of *Mycobacterium tuberculosis* lineage 2

*Mycobacterium tuberculosis* has several distinctive genetic features compared to other bacterial species, such as the extremely rare events of horizontal gene transfer (HGT), which leads to a clonal and hierarchical population structure (Boritsch et al., 2016). The underlying mechanism of rare HGT events in MTB is still under discussion. However, features of the genome such as the similarity of phylogenetic trees constructed by different genetic markers, as well as the constantly high G + C content in the genome and the rare occurrence of homoplasy, all indicate that MTBC has partially lost the ability for ongoing genetic recombination (Saelens et al., 2019). Given this structure, the evolution of *M. tuberculosis* depends on mutations, such as substitutions, deletions, and duplications of single nucleotides (Karmakar et al., 2019). Analysis of genotypes can provide unique information on the pathogen’s distribution dynamics and evolutionary genetics. Assessing the genetic distances between strains is used to complement epidemiological transmission data.

### 2.1 Genotyping methods

**Figure 1:**
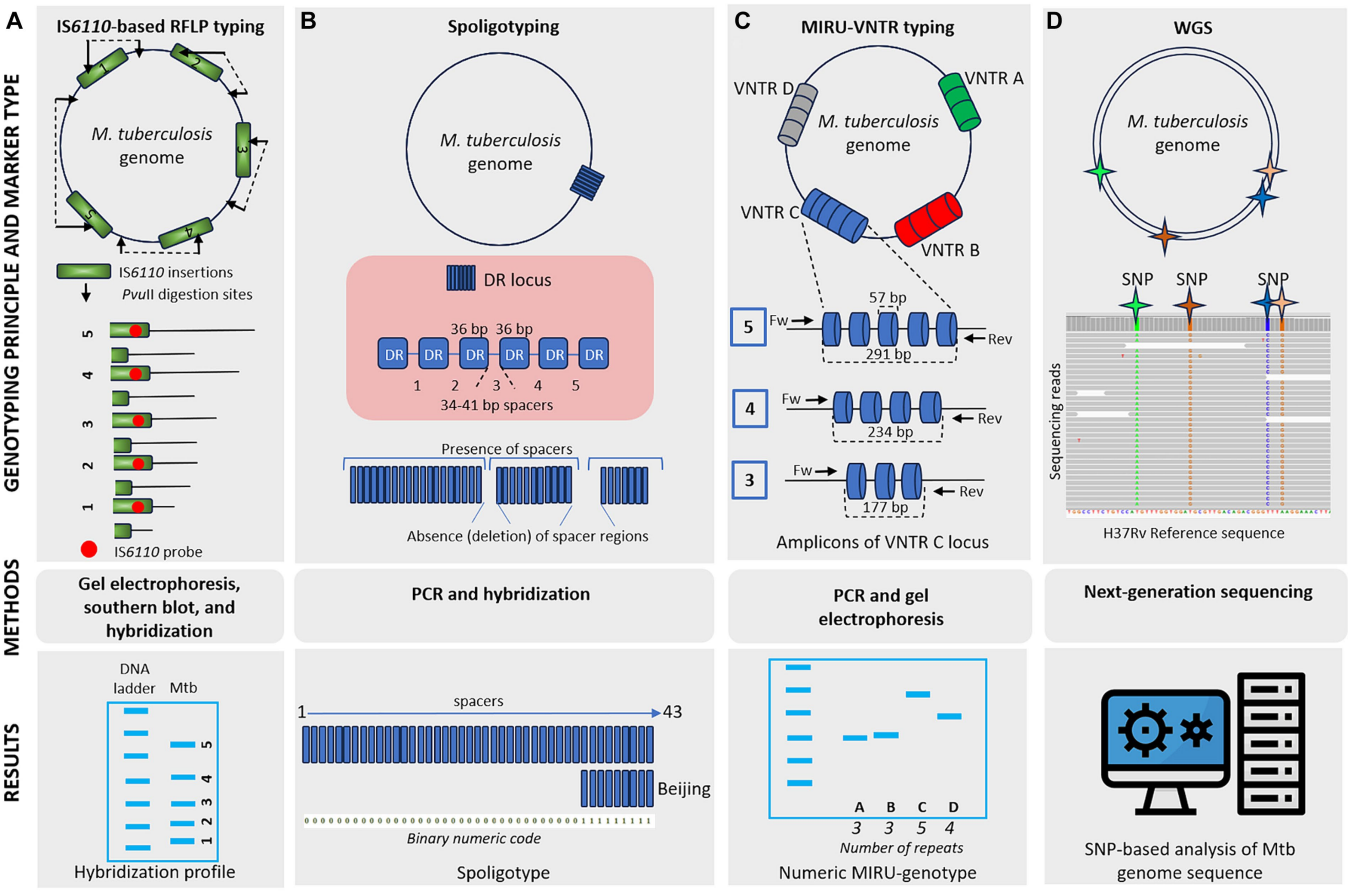
Schematic representation of different *M. tuberculosis* marker types and typing methods. **(A)** IS*6110*-based RFLP typing involves the cleaving of mycobacterial DNA with *Pvu*II, generating fragments of varying sizes. These fragments are separated by gel electrophoresis, transferred to a membrane, and hybridized, producing a banding pattern where each band represents a single IS*6110* element with flanking DNA of different lengths. IS*6110*-based RFLP typing offers high discriminatory power, although it is labor-intensive and requires skilled technicians. **(B)** Spoligotyping relies on PCR amplification of a single direct repeat (DR) locus with unique 34–41 bp spacer sequences. Genetic diversity depends on the deletion of these spacer regions. Hybridization of PCR products to a membrane containing oligonucleotides corresponding to spacers generates a pattern of positive or negative signals. Spoligoprofile and the binary code of the Beijing family *M. tuberculosis* strain is shown as an example. Spoligotyping is relatively simple and offers high-throughput capability, but it has limited discriminatory power compared to other methods and may not be suitable for all strain types. **(C)** MIRU-VNTR typing involves the amplification of variable number tandem repeat (VNTR) loci of different repetitive numbers scattered in the genome. PCR products are sized on agarose gels to determine the number of repeats at each locus resulting in a numeric MIRU-genotype. MIRU-VNTR typing provides higher discriminatory power than spoligotyping and allows for simultaneous analysis of multiple loci. **(D)** Whole-genome sequencing (WGS) utilizes information from the entire genome sequence, providing high-resolution data for SNP-based analysis. While WGS offers unprecedented resolution and accuracy, it is costly and requires advanced bioinformatics expertise.

**Figure 2:**
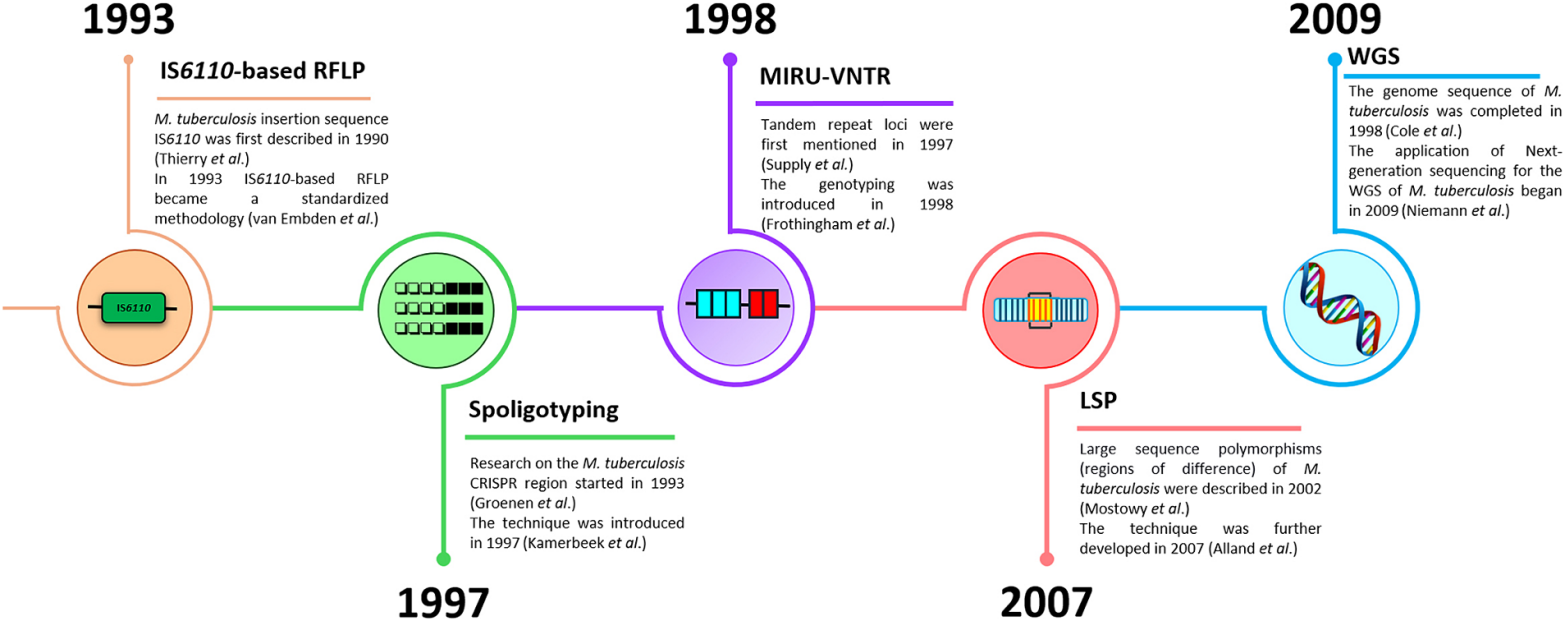
A timeline of *M. tuberculosis* genotyping methods (IS*6110*-based RFLP, spoligotyping, MIRU-VNTR, LSP, and WGS).

Genetic typing for molecular identification of clinical strains of *M. tuberculosis* has been widely used in recent decades. This molecular identification raised questions about strain-specific differences in clinical presentation and epidemiological characteristics of the infection. All MTBC genotyping methods can be classified based on repetitive sequences (IS*6110*, spoligotyping, and MIRU-VNTR), large sequence polymorphism (LSP) typing, and whole genome sequencing (WGS) for SNP-based analysis (Shevtsov et al., 2013; Jagielski et al., 2014; Żukowska et al., 2023). A schematic representation and timeline of *M. tuberculosis* marker types and genotyping methods are shown in Figures 1, 2.

#### 2.1.1 IS*6110*

The insertion sequence IS*6110* is a hallmark of the mycobacterial genome and became the first gold standard for strain genotyping. The insert is a fragment 1,361 bp long inserted into various parts of the genome and surrounded by inverted repeats 28 bp long. The IS*6110* RFLP detection assay itself is based on differences in copy number and identification of the insertion site in the genome. The introduction of IS*6110* into the genome is a random event that creates differences between tuberculosis strains both within and between families. The Euro-Asian lineage is characterized by a high copy number of this insertion, which may be associated with its influence on the evolution and virulence of strains of this lineage (Alonso et al., 2013).

Strains of the Beijing family showed similar patterns and were considered a homogeneous family based on IS*6110* profiles. Initially, two insertions were found: at the *dnaA-dnaN* origin of replication of the *M. tuberculosis* chromosome and one or two IS*6110* insertions (two for the W strain) in the NTF region (Bifani et al., 2002). Currently, IS*6110*-RFLP typing is not as common as before, however, IS*6110*-RFLP profiles have been accumulated in various databases (RIVM in the Netherlands), or PHRI in the USA and can be used to track strains in historical collections, which is compatible with more modern methods such as WGS (Mokrousov et al., 2021). However, the specific insertion site of this element in the Rv*2180c* gene is used to identify the Beijing family using PCR among clinical strains (Pérez-Lago et al., 2019). Despite its successful application in studying the epidemiology and spread of tuberculosis, this method has limitations, such as the requirement of large quantities of DNA with high concentration and purity. It is worth adding that the method is difficult to reproduce in different laboratories, as it is technically complex and does not discriminate sufficiently for isolates containing less than five copies of the IS*6110* element or none at all (Mestre et al., 2011; Coscolla and Gagneux, 2014).

#### 2.1.2 Spoligotyping

Spoligotyping is a method for typing the DR region, which is structurally composed of 43 spacers, each of which is surrounded by direct repeats. Identification is based on the absence or presence of a particular spacer sequence (Coscolla and Gagneux, 2014). The spoligoprofile of the L2 family lacks spacers 1 to 34, and at least three spacers 35 to 43 are observed (Shitikov et al., 2017). Spoligotyping is a cost-effective method for mycobacterial strains but has limited ability to discriminate sublineages of the L2 family. According to the SITVITWEB database, the spoligotypes of the L2 family are divided into SIT523 (777777777777771) known to be characteristic of proto-Beijing, and SIT1 (000000000003771), attributed to the Beijing family (Demay et al., 2012). However, this method is not always suitable for the other genetic families of MTB, such as the LAM family of Lineage 4. For instance, both SIT254 and SIT266 spoligotypes belong to the LAM family. However, in the SITVITWEB database, they are labeled as T5-RUS1 or T1, respectively. Notably, profiles SIT37 and SIT40 (T3 and T4 in SITVITWEB, respectively) have a single deleted spacer, yet they share the same family signature—spacers deleted from 33 to 36. Both of these WGS-based profiles demonstrate potential instances of convergent evolution, where spacer removal independently occurred in different isolates. Thus, it is recommended to be cautious with the interpretation of LAM spoligotypes and to complement spoligotyping with other genotyping methods (Mokrousov et al., 2017; Mokrousov, 2018).

#### 2.1.3 MIRU-VNTR

MIRU profiles identify polymorphic tandem repeat variations in *M. tuberculosis* chromosomes, which classify strains based on the number of repeats at different VNTR loci (Weniger et al., 2010). There are studies based on 12, 15, and 24 loci; therefore, typing at 15 and 24 loci has a higher discriminatory power compared to the classical method at 12 loci, especially for the Beijing family (Mokrousov, 2015; Shi et al., 2018). East Asian lineage genotypes can also be identified based on analysis of 24 loci using the online tool MIRUVNTRplus^1^ (Shitikov et al., 2017). In addition, according to the provided protocol, MLVA (Multiple Locus) VNTR subtyping is carried out based on the variable copy number of tandem repeats. Accordingly, a specific genotype is assigned to facilitate data classification. For example, the L2 family following MIRU-VNTR exhibits a wide copy number range, and MLVA analysis provides insight into clusters within the family at the microevolutionary level. Thus, for each pattern, a unique haplotype MtbC 15–9 is assigned and subsequently divided into genotypes/clusters and look like a set of numbers, so the Beijing family is characterized by the following clusters 94–32, 100–32, etc. (Weniger et al., 2010; Engström et al., 2019).

#### 2.1.4 LSP

Large sequence polymorphisms (LSPs) or regions of differences (RDs) are considered ideal phylogenetic markers since horizontal gene transfer rarely occurs in mycobacteria, making them irreversible (Coscolla and Gagneux, 2014). The classification is based on the presence or absence of 15 standard LSPs, which range in size from approximately 200 to 11,000 base pairs. For example, L2 is defined as the presence/deletion of five large genomic deletions (RD105, RD207, RD181, RD150, and RD142). Thus, deletions RD105 and RD207 are markers for the East Asian family, the latter leading to the absence of the first 34 spacers in the DR region. Further, proto-Beijing (RD105 deletion) and two Beijing lineages are divided into sublineages: 1 is Ancestral Beijing (RD105 and RD207 deleted) and 2 is Modern Beijing (RD105, RD207, RD181 deleted) (Cerezo-Cortés et al., 2019). It should be noted that RD markers initially do not differentiate between ancient and modern Beijing, this was achieved using whole genome sequencing (WGS) (Shitikov et al., 2017).

#### 2.1.5 SNP-based classifications

With the development of WGS methods, comparative analysis of genomes has identified sets of phylogenetically significant SNPs, which have been applied to the development of various strain typing methods. WGS has significant discriminatory power because it examines large regions of the genome (or whole genome) and SNPs exhibit very low degrees of homoplasy. The main types of SNP-based classification will be discussed in the next part of the review article. However, before the widespread use of this method, individual regions of the genome were studied and primers were selected to classify the main lineages of *M. tuberculosis*; for example, for L2, an SNP was identified at position Rv3304966 GA (position in reference genome H37Rv) (Fenner et al., 2011; Stucki et al., 2012). Another global classification of *M. tuberculosis* based on SNP divided them into six SCGs (SNP cluster groups), the Beijing family belonged to the SCG2 group (Filliol et al., 2006).

The division of isolates into Ancestral and Modern Beijing is significant for L2, this SNP is located in the MUT genes encoding DNA repair enzymes called ogt, mutT2, and mutT4. Thus, isolates of the ancestral family have *mutT2* mutation in codon 58 (GGA to CGA), causing the substitution of glycine by arginine, and the *mutT4* mutation in codon 48, causing the substitution of arginine by glycine (Rad et al., 2003). A more refined classification has been proposed based on polymorphisms of genes involved in DNA replication, recombination, and repair (3R). The complete differentiation scheme of the Beijing family divided them into 26 types of Bmyc sequences (Mestre et al., 2011).

Importantly, all proposed SNP-based classifications were consistent with the evolutionary pathways proposed by the other genetic markers described above. Despite this, these methods have limitations in distinguishing genotypes that do not always reflect actual phylogenetic groups, since deep genetic differentiation is only accessible and resolvable with WGS (Thawornwattana et al., 2021). However, it should be mentioned that classical methods are widely used in modern epidemiology, in particular, in the study of localized outbreaks of tuberculosis infection.

Portable long-read sequencing technology, such as the Nanopore platforms from Oxford Nanopore Technologies (ONT), provide an advantage over short-read sequencing methods, particularly in the case of the *M. tuberculosis* genome. This genome is characterized by extensive repeating elements, which pose challenges for short-read sequencing (Dippenaar et al., 2022). Furthermore, the TB Oxford Nanopore Diagnostics test has been developed to effectively detect drug resistance following tuberculosis diagnosis, facilitating clinical decisions regarding the treatment of drug-resistant tuberculosis. The analysis includes the simultaneous sequencing of amplicons from 27 targets, including 24 genes associated with drug resistance, the *hsp65* gene for identifying non-tuberculous mycobacteria, the DR genotyping target, and an internal control (World Health Organization, 2024).

## 3 Genomic diversity

The study of the genomic diversity of *M. tuberculosis* is characterized by the absence of horizontal gene transfer and is characterized by a strictly clonal population structure. Whole-genome sequencing methods are often used in studies of genomic diversity. To date, accumulating mycobacterial WGS data provides a phylogenetically robust basis for strain differentiation, coupled with resistance studies, providing information to quantify genomic diversity within or between groups of strains (Coscolla and Gagneux, 2014).

### 3.1 Lineage 2 classification

Based on techniques such as spoligotyping and RFLP, the Beijing family has been shown to lack genetic diversity and form a relatively recent clonal population. However, WGS analysis revealed that the family has evolved into several distinct sublineages over the past 1800 years (Luo et al., 2015; Liu et al., 2018a). Thus, based on the data, we know that *M. tuberculosis* is divided into 7 lineages, and the Transposon-derived region 1 (TbD1) deletion divides the species into modern and ancient representatives. This deletion is associated with modern strains of MTBC including L2 (East Asian), L3 (East African-Indian), and L4 (Euro-American), while evolutionarily “ancient” strains do not have this deletion (Brosch et al., 2002). The ancient lineages are L1 (Indo-Oceanic), L5 (West African 1), L6 (West African 2), and L7 (Ethiopian) (Comas et al., 2013).

The L2 lineage is in turn subdivided into two large-scale phylogenetic sublineages: proto-Beijing (L2.1) and Beijing (L2.2) (Shitikov et al., 2017; Ajawatanawong et al., 2019). The subsequent division of the L2 lineage is controversial in the scientific community. The main difficulty is the hierarchical population structure of the mycobacteria, which consists of large phylogenetic lineages, smaller sublineages, and, finally, clonal clusters. Thus, in this review, we will focus on the main proposed classifications.

The classification proposed by Coll et al. based on SNPs and consistent with the RD differentiation system, divides *M. tuberculosis* into 7 lineages and 55 sublineages. Interestingly, the phylogenetic analysis in this study revealed the presence of new clades when RD was not able to differentiate them. Thus, in addition to the L2-specific deletion RD105, other deletions (RD207, RD181, RD150, and RD142) were discovered within the L2.2 lineage at the microevolutionary level (Coll et al., 2014). Despite the low Hunter Gaston discriminatory index (0.18), the proposed classification was adapted to changes and the addition of new genotypes (Shitikov et al., 2017).

Further, Merker et al. classified the L2.2 lineage (Beijing) into eight main family sublineages, the scheme included three ancient groups, known as Asia Ancestral 1, 2, 3 and five modern lineages: Asian African 1, 2; Pacific RD150; Europe-Russia B0/ W148 and Central Asia. These phylogenetic branches of the L2 family were constructed based on SNPs. Six sublineages or clonal complexes (CC) based on 24 MIRU-VNTR markers were also established: CC1, CC2, CC3, CC4, CC5, and CC6, and the basal subline BL7 was isolated separately. A subsequent study of the NTF region for the IS*6110* insertion among the selected 337 strains divided CC into two groups, so CC1-CC5 belong to modern isolates, and the remaining CC6 and BL7 include atypical ancestral Beijing variants (Merker et al., 2015). The proposed classification is considered incomplete for the L2 lineage since it does not include the evolutionary subgroup L2.1. However, the Hunter Gaston discrimination index (0.77) has good discriminatory ability (Shitikov et al., 2017).

**Figure 3:**
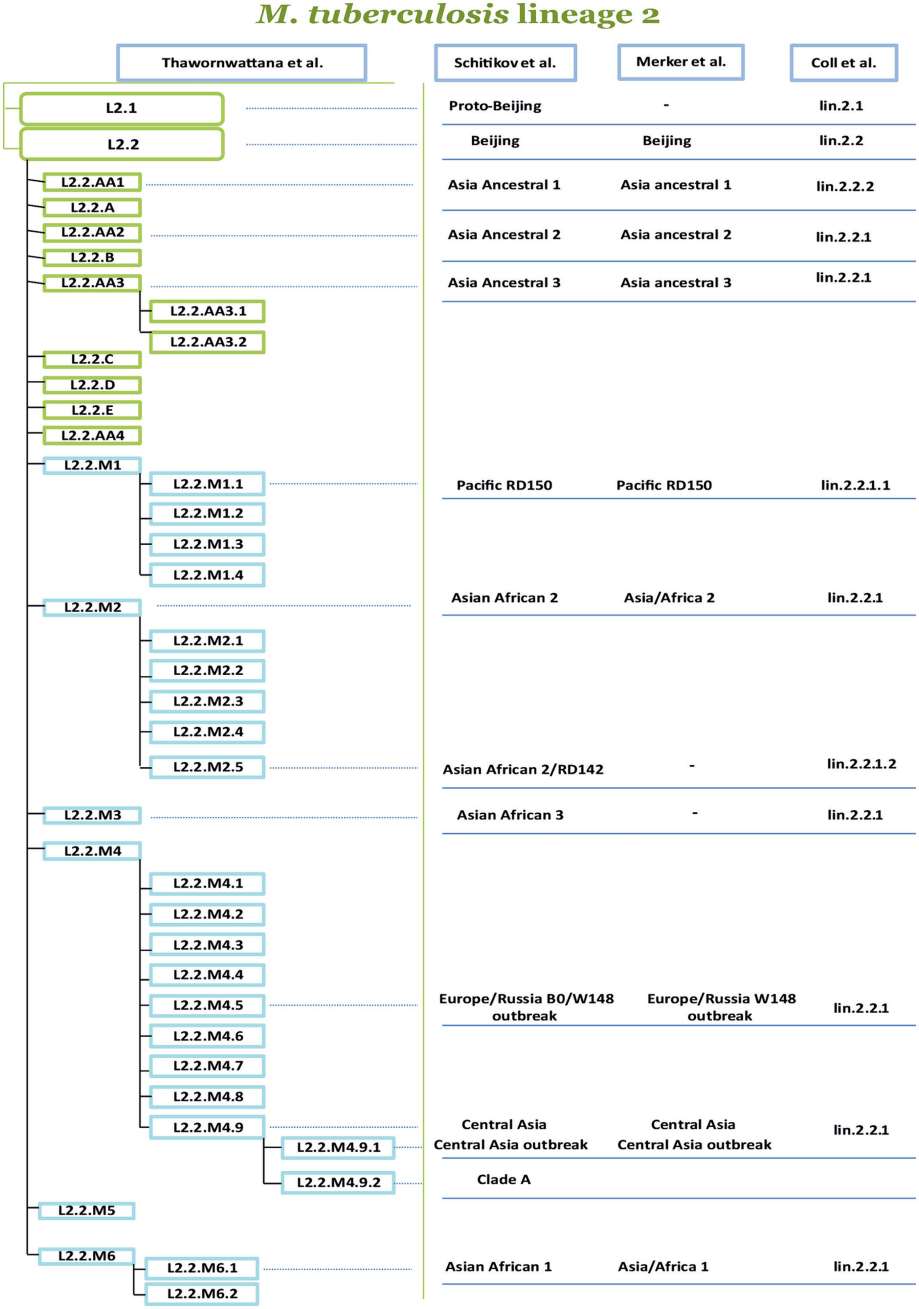
Comparison of four phylogenetic classifications of *M. tuberculosis* lineage 2. The authors of the proposed nomenclatures are indicated at the top of the table, from left to right (Coll et al., 2014; Merker et al., 2015, 2018, 2022; Shitikov et al., 2017, 2019; Thawornwattana et al., 2021). The most comprehensive classification is proposed by Thawornwattana et al. is divided into several levels, the level-1 is L2, the level-2 is L2.1, and L2.2. The level-3 divides L2.2 into ancient (A) and modern (M), and level-4 and level-5 represent subsequent genetic clusters. The other classifications of the sublineages and clusters from Shitikov et al. (2017, 2019), Merker et al. (2015, 2018, 2022), and Coll et al. (2014) are connected by lines dash-dotted line with labels from the classification of Thawornwattana et al. (2021).

Another classification was proposed by Shitikov et al., which had higher discriminatory power (HGDI 0.79), as it considers two sub-lineages: L2.1 and L2.2. In turn, the Beijing group (L2.2) consists of 10 groups, three of them belong to the “ancestral” Beijing group (ancestral Asia 1, ancestral Asia 2, ancestral Asia 3), and seven belong to the “modern” Beijing group (Asiatic-African 1, Asia-Africa 2, Asia-Africa 2/RD142, Asia-Africa 3, Pacific RD150, Europe/Russia B0/W148 outbreak and Central Asia). The proposed classification system summarized the SNPs of several studies, and sets of markers were studied to construct a phylogenetic tree of L2 strains (Shitikov et al., 2017). Recently Thawornwattana et al. proposed a classification scheme complementing Shitikov et al., and expanding the L2.2 sublineage. Currently, it is the most complete classification in terms of genetic diversity covering various endemic regions. A classification with a total of 15 genotypes and three levels was proposed, with genotype designations for strains following a scheme similar to Rutaihwa et al. (2019). In addition to numbers, letters were added to the classification; for Ancestral the letters from “A” to “E” were used and for Modern Beijing, it was designated “M.” In the proposed classification, it is worth noting the revised Ancestral Beijing genotypes and the proposed six Modern Beijing genotypes (L2.2.M1–L2.2.M6). The revised nomenclature of L2 proposed by Thawornwattana et al. is currently the most comprehensive in comparison with the other known classifications (Figure 3). Figure 3 is based on the Thawornwattana et al. classification scheme of L2, each branch in the nomenclature corresponds to specific SNPs, which are utilized as SNP barcodes. In summary, the proposed classification is easy to interpret, compatible with the known genotypes, and expandable if novel genotypes are discovered (Thawornwattana et al., 2021).

The genomic diversity of *M. tuberculosis*, characterized by a strict clonal population structure and the absence of horizontal gene transfer, plays a key role in the formation of phylogenetic relationships. However, in the case of modern Beijing tuberculosis strains, phylogenetic relationships pose a challenge because the star-shaped phylogeny leads to the independent evolution of new groups. This situation causes controversy and inconsistency between different authors, leading to the proposal of different genotyping schemes in attempts to elucidate evolution, and the diversity of groups depends on the collection of strains. Therefore, newly developed classifications should not ignore previous ones, since this issue is extremely important for public health.

### 3.2 Beijing B0/W148 and Central Asia outbreak

The phylogenetic division of modern Beijing family strains into clonal clusters has clinical and epidemiological significance. These strains were attributed to be associated with outbreaks of multidrug-resistant tuberculosis (MDR-TB) and characterized by high mutagenic potential, as well as increased transmissibility and hypervirulence (Merker et al., 2018). Distinctive outbreaks circulating in Russia and Central Asia include the following strains: B0/W148, and Central Asia outbreak (CAO) (Shitikov et al., 2017; Merker et al., 2018; Akhmetova et al., 2023). According to the nomenclature of Thawornwattana et al., the outbreaks are characteristic of the L2.2.M4 lineage, and in particular L2.2.M4.5 for B0/W148 and L2.2.M4.9.1 for CAO (Thawornwattana et al., 2021).

The origin of these strains has always been of interest, so initially there was a hypothesis about the emergence of Beijing B0/W148 in Siberia, with subsequent spread in 1960–1980 throughout Russia, mainly to the European part of the country (Mokrousov, 2013). Later, Merker correlated the strains with clonal clusters according to MIRU-VNTR. Accordingly, the 94–32 Central Asian (or Central Asian/Russian) cluster including CAO was assigned to the CC1 complex, while the 100–32 cluster including B0/W148 genotype was assigned to the CC2 complex, dominant in Russia and Eastern Europe. Presumably, the most recent expansion of these genetic lineages occurred around the beginning of the 19th century, which may be associated with the national uprising in China from 1861 to 1877, which caused population migration to the Russian Empire, especially to the countries of Central Asia and the spread of CC1 strains and CC2 in this region (Merker et al., 2015). The time of origin was confirmed in a study by Shitikov et al. when they analyzed clades B (part of the B0/W148 outbreak), A and CAO and concluded that these lineages formed approximately 180 years ago (Shitikov et al., 2017). It is worth noting that it is the MDR strains of these clusters that have spread recently to the territories of Russia, Kazakhstan, Kyrgyzstan, and Uzbekistan. The distribution of clonal complexes was accelerated by the disruption of the public health system during the collapse of the USSR (Merker et al., 2015).

Recent studies suggested that the B0/W148 strain appeared in Central Asia in the early 60s of the 20th century and subsequently spread westward, with epidemic waves continuing until the end of the 20th century. Analysis of genetic diversity revealed a similar genetic profile and little variation with a mean of 32 SNPs between strain samples, supporting the idea of their close relationship and therefore outbreak clone status. Thus, the successful spread of this clone was provoked by two factors, namely, the genetic characteristics of the mycobacteria and the impact of historical and political events. Thus, the spread began in the late 70s, followed by a 20-fold increase in the population over 10 years (Merker et al., 2022).

The CAO outbreak is similar in timing to B0/W148, with a lineage beginning in 1974, with the highest probability (95% confidence interval) occurring between 1969 and 1982 (Merker et al., 2018). This clade dominates the territory of Central Asian countries, and also became widespread and acquired MDR in the 90s during the collapse of the USSR (Merker et al., 2018; Klotoe et al., 2019). According to the phylogenetic relationship, it descends from the Central Asian (CA) sublineage L2. Since these clades are of epidemic importance in healthcare, PCR test kits were developed for their rapid detection (Mokrousov et al., 2012; Shitikov et al., 2019).

The genetic diversity of *M. tuberculosis* is represented by 7 lineages. L2 shows a complex phylogenetic substructure and forms star-shaped structures on the tree clusters. This structure causes many controversial issues in the classification of isolates, and it is important for determining epidemiological studies, controlling the spread of the disease and developing effective treatment strategies. In particular, *M. tuberculosis* strains belonging to the B0/W148 and Central Asia outbreak (CAO) clades are associated with outbreaks of multidrug-resistant tuberculosis. Similarities in the evolution and genetic characteristics of these strains highlight the importance of historical events and genetic features in their distribution and epidemiological significance.

## 4 Geographic distribution

A study of population genomics and genotyping data identifies several hypotheses for the geographical origin of lineage 2, the first suggests that it has originated in the northeastern region of Asia, and the other suggests its emergence in the southern territory of East Asia with subsequent spread to the South (Mokrousov, 2008; Merker et al., 2015; Yin et al., 2016). It is important to note that there is a growing body of research supporting the assumption of a Southeast origin for the Beijing family (Luo et al., 2015; Guyeux et al., 2022). This conclusion is justified by the high genetic diversity and wide distribution of this family in this region. The case for a southern Chinese origin is strengthened by recent observations of the L2 sublineage described as “Asian Ancestral 4” or L2.2.AA4. This study was conducted in northern Thailand, Chiang Rai, where ethnic minorities have lived since the 7th century. The tribes migrated from southern China to this province about 100 years ago, so this sub-lineage has connections to this region, highlighting a possible southern Chinese origin. It has also been hypothesized that the evolutionary transition from ancestral to modern Beijing sublineages occurred in Southern China. This connection is indicated by the presence of a mutation in Ancestral 4 or L2.2.AA4, which is characteristic of modern Beijing samples (Ajawatanawong et al., 2019). It is worth noting that the origin of the ancestral lineage known as Proto-Beijing also extends to southern China (Luo et al., 2015). Evidence for a northern origin of the family is reflected in a recent study, where strains of the L2.3 (Modern Beijing) or L2.2.M lineage were shown to originate from northern East Asia (Zhu et al., 2023).

The temporal origin of the L2 lineage has been widely speculated; the age of the mycobacteria is 70,000 years, and the L2 lineage is 30,000 years old (Comas et al., 2013; Luo et al., 2015). Sublineage analysis revealed that the time of fusion of two genetic lineages, namely proto-Beijing and ancestral Beijing, occurred approximately 2,200 years ago. The time of separation of all of Beijing’s ancestral lineages was also estimated to be approximately 1,300 years ago. Within these estimates, the expansion of Proto-Beijing was estimated to have occurred approximately 900 years ago, while the emergence of modern Beijing was dated to approximately 500 years ago (Liu et al., 2018a). However, analysis of only the L2.2 sublineage revealed a time of 6,000 years before the common ancestor of L2 (Merker et al., 2015). The estimated time of appearance of the most recent common ancestor of modern Beijing specimens, or L2.2.M, occurred 591 years ago. It is assumed that the sublineage arose approximately 1,200 years after the appearance of its ancestor (Zhu et al., 2023). These time estimates help to better understand the evolutionary chronology and genetic dynamics of L2 lineages, as well as their relationship to the history of migrations and interactions of human populations.

**Figure 4:**
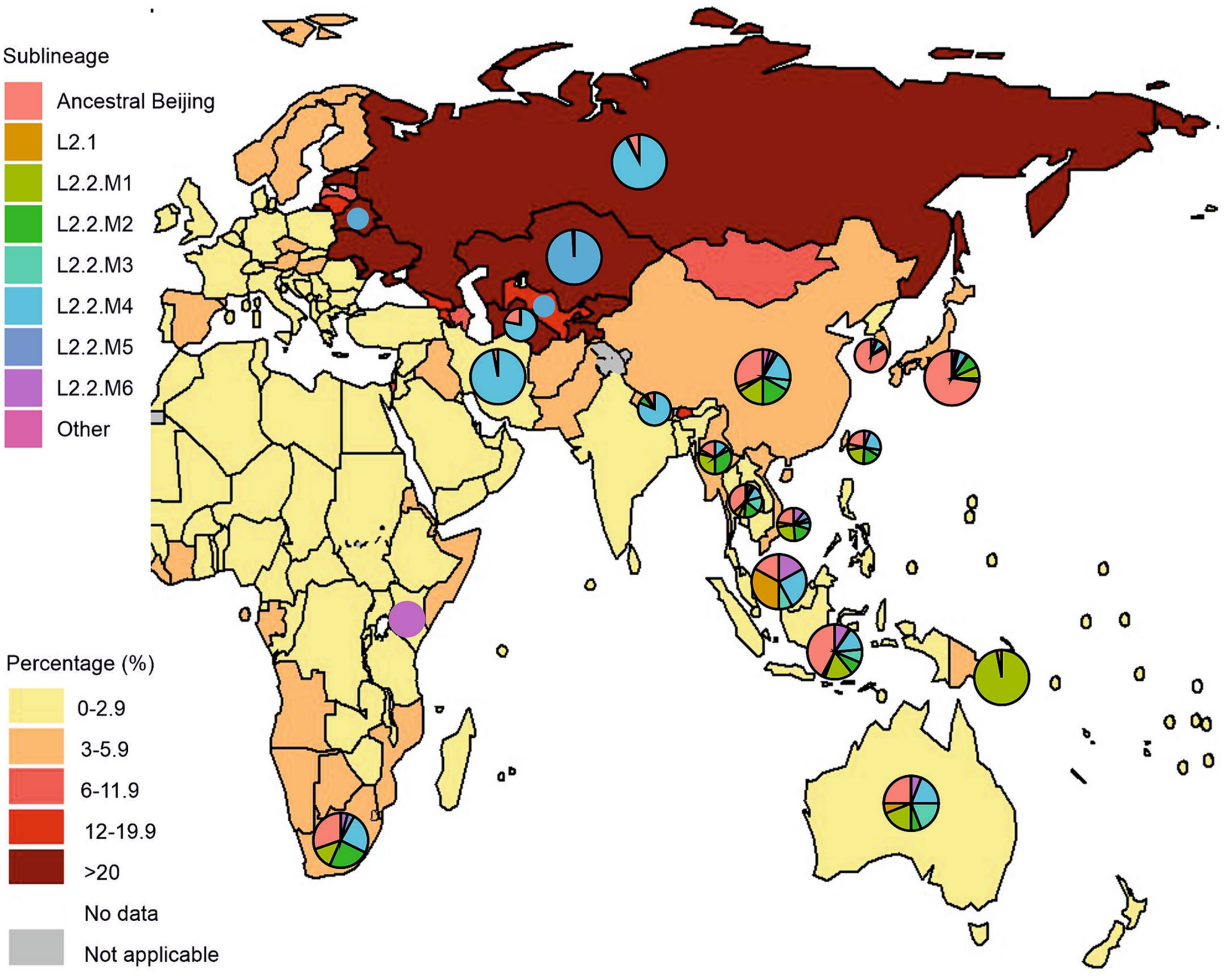
Geographical distribution of *Mycobacterium tuberculosis* lineage 2 and new cases of tuberculosis with MDR/RR-TB by country and region. Pie charts represent a distribution of L2 sublineages positioned over countries with a high prevalence of L2 isolates, mostly from Asia, Eastern Europe, Oceania, and East/Southern Africa. Pie sizes are not proportional to the number of isolates from each location. The figure was adopted from Figure 2.3.6 “Percentage of new TB cases with MDR/RR-TB, 2021” from the Global Tuberculosis Report 2022 (Geneva: World Health Organization; 2022). The countries with a high prevalence of L2 were selected based on the comprehensive systematic analysis by Wiens et al. (2018) representing over 200,000 bacterial isolates collected over 27 years in 85 countries. The list of countries with a high prevalence of L2 and the distribution of sublineages is represented in Supplementary Table S1.

Human-adapted *M. tuberculosis* complex (MTBC) lineages exhibit geographic specialization. Lineages L2 and L4 are widely distributed throughout the world, representing “widespread” lineages, while L5, L6, and L7 are geographically restricted and are “regionally restricted” lineages (Coscolla and Gagneux, 2014; Tarlykov et al., 2020). There has been geographic variability in the distribution of L2 strains to date. In some countries, certain sublineages or even genetic clusters predominate (Figure 4). For example, the overall prevalence of lineage 2 in the East Asia region reaches 60%. This geographic dispersion of L2 sublineages is aggravated by the high prevalence of MDR tuberculosis. Figure 4 visually supports a strong positive correlation between a prevalence of the L2.2.M4 sublineage and a higher proportion of new TB cases with MDR/RR-TB in Russia, Belarus, and Central Asian countries (Kazakhstan, Uzbekistan, and Turkmenistan). Moreover, L2.2.M4 sublineage is known for two MDR-TB outbreak strains of the L2.2.M4.9 Central Asian clade: Clade A and Central Asia Outbreak (CAO), as well as L2.2.M4.5 (Europe/Russia B0/W148) and L2.2.M4.1 (Bmyc22) outbreak, making it a significant public health concern.

L2.1 (proto-Beijing) is primarily composed of isolates originating from China, Thailand, and Vietnam, with a smaller number from Japan, Malaysia, and Indonesia. Notably, there is not a clear delineation by country, although a distinct cluster from Vietnam suggests the emergence of a local strain. Generally, isolates from China, Thailand, and Vietnam are distributed relatively evenly throughout the phylogeny, spanning across most L2 sublineages. This suggests their longstanding presence and hints at the potential origin and diversification of L2 strains within the region.”(Thawornwattana et al., 2021). L2.1 strains do not exhibit distinct clinically significant features and are characterized by moderate transmission capacity, as is the case in Japan (Guyeux et al., 2022). However, a study by Mokrousov et al. found two clusters of the ancient Beijing sublineage in the Russian part of Asia, and, importantly, these strains were multidrug-resistant (Mokrousov et al., 2019, 2023). In contrast, modern sublineages of the Beijing family show a global distribution in various regions of the world (Figure 4). This indicates the high adaptability of modern Beijing sublineages in the context of global mycobacterial dynamics (Luo et al., 2015; Liu et al., 2018a).

According to a study by Merker et al. (2015), the following Beijing CCs exhibit different geographic distribution patterns: CC1 (Central Asia), CC2 (Russia and Eastern Europe), CC3 (East Asia, the Pacific, and the Americas), CC4 (East and Southern Africa and Pacific), CC5 (Pacific, Micronesia and Polynesia), CC6 and BL7 (East Asia, North America and Mexico). The geographic distribution of CCs has been confirmed by several studies. Thus, in East and Southern Africa there are “modern” Asian-African Beijing sublineages (L2.2.M2, L2.2.M3, L2.2.M6.1) and “ancient” Beijing strains (L2.2.AA1, L2.2.AA2, L2.2.AA3). However, strains CC1 and CC2, characteristic of Eastern Europe and Central Asia, are completely absent in African populations. This indicates their relatively recent origin and their likely formation outside East Asia (Rutaihwa et al., 2019).

Overall, the L2.2.M1 clade is represented widely across geographic regions, evenly distributed across the countries of China, Japan, South Korea, Taiwan, Vietnam, Thailand, Myanmar, Indonesia, and Australia. This clade is also characterized by local outbreaks in Papua New Guinea and South Africa. L2.2.M2 consists of five subclades designated L2.2.M2.1 – L2.2.M2.5, L2.2.M2.4 is a small group consisting of isolates originating from China, and the remaining four clades are widespread in China, Vietnam, Thailand, and South Africa. It is worth noting that this clade is dominant in Myanmar. The L2.2.M3 clade is distributed evenly in the countries of China, Taiwan, Vietnam, Malaysia, and Indonesia. Interestingly, isolates from Thailand and Australia dominate the number of samples in this cluster. Thus, the Thai samples form a separate subcluster, which is associated with the recent outbreak of MDR in Thailand (Thawornwattana et al., 2021).

The L2.2.M4 clade consists of nine subclades and is expressed by the distribution of clusters across geographic regions, so L2.2.M4.1 and L2.2.M4.2 are found mainly in Thailand. In China, L2.2.M4.3, L2.2.M4.6, and L2.2.M4.8 predominate; L2.2.M4.4 is mainly from South Africa, and L2.2.M4.7 is from Nepalese isolates (Thawornwattana et al., 2021).

In the countries of Central Asia, Eastern Europe, and Russia, L2 strains dominate, mainly the Modern Beijing sublineage, such as L2.2.M.4.5 (B0W/148, Clade B), L2.2.M4.9 (Central Asian), L2.2. M4.9.1 (CAO) and L2.2.M4.9.2 (Clade A) (Ibrayeva et al., 2014; Shitikov et al., 2017; Auganova et al., 2023). At the country level, the highest proportions of new TB cases with MDR/RR-TB were found in the Russian Federation, Central Asia, and several countries in Eastern Europe. Russia was the transboundary area for these clades to Europe and Central Asia, with Eastern Europe a possible center of dispersal (Zhu et al., 2023). At last, L2.2.M5 and L2.2.M6 are small clades formed recently and represent samples from Vietnam, Thailand, and China, in addition, L2.2.M6 are found in Kenya and South Africa (Thawornwattana et al., 2021).

### 4.1 Transmission dynamics

Although MTBC lineage 2 is represented by sublineages predominantly in certain geographic regions, the global distribution of these strains suggests its high transmission potential in different parts of the world. Previously thought to be a family-wide occurrence of increased transmissibility, such transmission is now known to be limited to the L2.2.M sublineage, and in particular to newly emerging clades within the sublineage (Liu et al., 2016; Zhu et al., 2023).

This increased transmissibility is partly the basis for the “MDR outbreak hypothesis” proposed by Merker et al. Thus, clusters 94–32 (CC1) and 100–32 (CC2) in a study of average pairwise genetic distances between genomes showed low values and fewer mutations, which confirms their relatively recent distribution in Russia and Central Asia. However, the spread of the two clusters occurred before the antibiotic era, which may indicate that the spread of drug resistance in these countries with shared genetic characteristics was not the underlying cause but was likely a consequence of broader problems in public health systems and clinical practice (Merker et al., 2015).

Intriguing data was shown by Zhu et al., when analyzing modern L2.2.M (L2.3) lineages from 51 countries, so according to their classification, 84.8% of strains belong to clades from L2.3.4 to L2.3.6. It is important to note that the “MDR outbreak hypothesis” clades belong to L2.3.6 and even after excluding them from the analysis, the percentage was higher than that of L2.3.1-L2.3.3 (Zhu et al., 2023). This shows that the spread of modern lineages occurred not only in this region but in connection with the B0/W148 and CAO outbreaks throughout the world.

High virulence in modern lineages is associated with positive selection in several genes, such as *ftsK, fadE17*, and, *Rv2209*, which led to their fixation in the population and likely provides adaptation benefits to lineage 2 (Zhu et al., 2023). Merker et al. also isolated the *kdpDE* operon, which is located inside the *Rv0176* gene, encoding a protein associated with MCE1 (molecular complex of extracellular pathogen 1). Thus, in strains of the B0/W148 (CC2) subline, a frameshift mutation was detected in this operon, which presumably may lead to increased virulence due to the formation of a fusion protein with a change in its functions (Merker et al., 2015). Subsequent study of the genetic characteristics of modern Beijing strains can serve as a model for understanding the dynamics of the pathogenicity of this family as a whole.

### 4.2 Drug resistance and compensatory mutations

Transmission-driven drug-resistant (MDR/XDR) tuberculosis stands as the foremost contributor to human mortality arising from antimicrobial resistance. For example, specific lineage 2 clades, such as B0/W148 or CAO, currently play a pivotal role in the elevated prevalence of MDR-TB in the Eurasian region (Merker et al., 2022).

Shortly after Isoniazid (INH) began to be used against TB in the 1950s, Middlebrook found that most INH-resistant MTB strains were less virulent in Guinea pigs (Middlebrook, 1954; Middlebrook et al., 1954). Based on these observations, it was believed that drug-resistant strains were inherently weaker, or less fit than drug-sensitive strains. In contrast, the situation with the drug-resistant strains was observed to be quite the opposite. One plausible explanation is the acquisition of compensatory mutations after the emergence of resistance mutations. Initial findings by Gagneux et al. emphasized the impact of drug resistance-associated mutations on the bacterial fitness of MTB strains (Gagneux, 2009; Emane et al., 2021). Over time, resistant mycobacteria accumulate additional mutations to offset the initially observed fitness deficits, thereby mitigating the reduction in overall fitness (Maisnier-Patin and Andersson, 2004; Bhatter et al., 2012; Abeldenov et al., 2015; Kodio et al., 2019; Daniyarov et al., 2023). Importantly, the acquisition of compensatory mutations following the introduction of resistance-conferring mutations, such as *rpoB* S450L, plays a crucial role in elevating transmission rates. It has been suggested that compensatory mutations are associated with structural changes in RNA-polymerase *rpoA, rpoB*, and *rpoC*, which increases the transcriptional activity, following the growth of MTB. The *rpoB* gene codes for the RNA polymerase β subunit, which is the target of Rifampicin (RIF), an important anti-tuberculosis drug.

Song et al. extensively documented the occurrence of compensatory mutations strongly linked to the *rpoB* S450L mutation in a population of MDR strains. Compensatory variants were identified in 54% of strains harboring S450L, in stark contrast to only 10% of isolates carrying other mutations in the *rpoB* gene (*p* < 0.0001) (Song et al., 2014). Numerous studies further reinforce a strong association between the RIF resistance-associated mutation S450L and compensatory mutations in *rpoABC* genes (Comas et al., 2011; Kozhamkulov et al., 2011; Li et al., 2016; Liu et al., 2018b; Bainomugisa et al., 2018). This correlation has been firmly established through diverse experimental approaches, including the generation of mutant MTB clones, assessment of growth rates in comparison to the drug-sensitive parent strain, and evaluation of their relative capacity to induce TB disease or mortality in animal models (Brandis and Hughes, 2013; Vos et al., 2013; Boritsch et al., 2016).

Merker et al. (2018) examined the evolutionary history, resistance, and transmission of MTB isolates on the territory of Uzbekistan in Central Asia. The CAO cluster accounted for about three-quarters of all MDR-TB isolates in the studied population (Merker et al., 2018). The existence of mutations that should compensate for fitness deficits was related to the transfer of drug-resistant TB. The average number of drug-resistance mutations was higher among the isolates with compensatory mutations. Moreover, the isolates with compensatory mutations demonstrated a higher ratio of transmission in comparison to the isolates with no compensatory mutations.

Usually drug-resistant strains are detected in a handful of patients and may be less fit than most drug-sensitive strains, however, regions with high rates of DR-TB show MDR burden caused by a few dominant, highly transmitted genetic clusters of closely related strains (Casali et al., 2014; Disratthakit et al., 2015). Globally, the estimated transmission of MDR-TB strains is responsible for over 70% of MDR-TB cases (Kendall et al., 2015). There is a possibility that differences in strain transmissibility and the equilibrium between the fitness costs associated with specific resistance-conferring mutations and the capacity of acquired compensatory mutations to reinstate the fitness of the resistant strains.

A recent investigation has illuminated the link between resistance mutations and their associated fitness costs with the strain genotype (Castro et al., 2020). *In vitro* studies reveal distinct frequencies of fluoroquinolone-resistant gyrase mutations in different strains of *M. tuberculosis*, each exhibiting preferences for specific mutations and varying fitness costs. Lineage-specific mutation preferences were observed in clinical strains, with fluoroquinolone-resistant strains of lineage 2 predominantly featuring the *gyrA* Asp94Gly substitution, while lineage 4 strains more frequently displayed the *gyrA* Ala90Val substitution. Notably, the complexity and variability of the acquisition and effects of these mutations are expected to be more intricate when they manifest in the diverse array of *M. tuberculosis* strains causing tuberculosis.

## 5 Conclusion

This review article concludes by emphasizing that *M. tuberculosis*, particularly, lineage 2, poses a serious public health threat. Experimental and clinical data confirm the high virulence and high transmissibility of these strains, especially as represented by the L2/Beijing family.

The article also examines the genomic diversity of *M. tuberculosis* and the unique genetic features of lineage 2. Currently, the WGS provides the most comprehensive approach for strain differentiation and assessment of genomic diversity. The complex phylogenetic structure and star-shaped structures (distribution of genotypes within a cluster where there is one main “center” of high frequency surrounded by less frequent variants) observed in L2, particularly the L2.2.M lineage, pose a challenge to accurately classifying isolates, which highlight the critical role of this lineage in epidemiological studies, especially in the context of multidrug resistance outbreaks caused by the B0/W148 or CAO clade. Drug resistance and the impact of compensatory mutations on the high transmissibility and virulence of lineage 2 are discussed.

The origin of lineage 2 in East Asia, as well as its distribution across various geographical regions, remains controversial and may be an important direction of future research. Another issue under consideration is a unified classification of lineage 2 that should be accepted globally to avoid misinterpretation of MTBC clusters and genotypes. Overall, the article aims to review the genomic characterization of lineage 2 strains, identifying key trends and prospects for future research.

## Author contributions

SA: Conceptualization, Visualization, Writing – original draft, Writing – review & editing. DA: Writing – review & editing. PT: Conceptualization, Funding acquisition, Writing – original draft, Writing – review & editing.

## Footnotes

^1^http://www.miru-vntrplus.org
